# Impact of COVID-19 on Otorhinolaryngologists in Bulgaria: A Survey of Healthcare Professionals

**DOI:** 10.7759/cureus.79229

**Published:** 2025-02-18

**Authors:** Kamelia Milcheva, Nikolay R Sapundzhiev

**Affiliations:** 1 Otolaryngology, Medical University of Varna, Varna, BGR

**Keywords:** bulgaria, covid-19, ent, otolaryngology, otorhinolaryngology

## Abstract

Background

The COVID-19 pandemic caused significant global social and economic disruption, highlighting the challenges of emerging infectious diseases. It also pushed modern medicine and healthcare systems to the wall. The mode of transmission of the SARS-CoV-2 infection is by aerosolized upper respiratory tract secretions. The otorhinolaryngology specialty seems to be among the riskiest for contracting airborne infections. This is because of the close face-to-face contact with the patients during routine examinations and many specific procedures. This study aimed to evaluate otolaryngologists’ self-assessment of their comparative infection risk and the impact of the COVID-19 pandemic on their clinical practice, financial stability, and psychological well-being.

Methodology

A national cross-sectional survey was conducted among otorhinolaryngologists in Bulgaria by a research team from the Medical University of Varna. The study assessed infection risk perception, changes in practice, psychological stress, and financial effects. Data were collected via Survs.com and analyzed using descriptive statistics, cross-tabulations, and chi-squared tests, with significance set at 0.05 in Jamovi version 2.3.28.

Results

Of the 241 invited participants, 120 (49.8%) completed the survey. In total, 61 (50.8%) perceived their infection risk as similar to emergency healthcare workers, while 91 (75.8%) likened their exposure to that of dentists. Workplace exposure was identified as the main infection source by 95 (79.1%), with 71 (59.1%) considering outpatient clinics the highest-risk setting. Financially, 47 (39.1%) reported no income change, 31 (25.8%) experienced reductions, and 42 (35.0%) saw increased earnings. A chi-square test revealed a significant association between financial impact and workplace setting (χ² (3) = 11.68, p = 0.0086). Hospital-based otolaryngologists were more frequently affected by income reductions, with 65 (54.2%) reporting financial losses compared to 22 (18.3%) in outpatient settings. The psychological toll was substantial, with 44 (36.6%) reporting high stress, 34 (28.3%) very high stress, and only 3 (2.5%) stating no effect. Significant associations were found between stress levels and workplace setting (χ² (4) = 12.4, p = 0.015) and work experience (χ² (12) = 22.9, p = 0.029).

Conclusions

The COVID-19 pandemic profoundly affected otorhinolaryngologists’ financial stability, work environments, infection risk perception, and mental health. Hospital-based specialists faced greater financial challenges, while stress levels were significantly linked to workplace setting and experience. These findings highlight critical areas for long-term changes in otorhinolaryngological practice.

## Introduction

The COVID-19 pandemic, caused by the novel coronavirus SARS-CoV-2, has deeply transformed healthcare practices globally. Otorhinolaryngologists faced unique challenges during the COVID-19 crisis due to their close contact with the upper airway, leading to significant changes in clinical practice. As the pandemic evolved, the effects of COVID-19 on otorhinolaryngology became increasingly evident, reshaping the way clinicians and patients interact. The mode of the SARS-CoV-2 infection is by aerosolization from upper respiratory tract secretions during examination and surgical procedures [1,2]. Among surgical specialties, otorhinolaryngologists are at a particularly high risk of COVID-19 exposure [3-6]. There is evidence that otorhinolaryngologists run a high risk of occupational SARS-CoV-2 due to high viral load related to upper aerodigestive tract examination and clinical procedures [5]. One of the first instances of COVID-19-related death among physicians during the recent pandemic was among otorhinolaryngologists, with Mr. Adil El Tayar and Mr. Amged El-Hawrani being among the earliest reported fatalities [7,8]. The same applies to SARS [6]. This study seeks to answer the following questions: (1) how do Bulgarian otorhinolaryngologists perceive their risk of COVID-19 infection? (2) What changes occurred in clinical practice and financial stability? (3) How did the pandemic affect psychological well-being?

## Materials and methods

Study design

This was a national cross-sectional survey-based study aimed at evaluating the impact of the COVID-19 pandemic on the otorhinolaryngological practice in Bulgaria. The study focused on understanding otorhinolaryngologists’ perceptions of infection risks, changes in practice, psychological stress, and financial outcomes during the pandemic. It was conducted over nine months (July 2022 to March 2023) by a research team from the Faculty of Medicine and the Faculty of Public Health of the Medical University of Varna and followed ethical guidelines approved by the Committee for Ethical Scientific Research at the Medical University of Varna (approval number: 118/23.06.2022). The Bulgarian National Society of Otorhinolaryngology, Head and Neck Surgery, further approved the study. Participation in the study was voluntary and anonymous.

Inclusion and exclusion criteria

Inclusion criteria were currently practicing otorhinolaryngologists in Bulgaria. Participants provided informed consent before accessing the survey. Exclusion criteria were incomplete survey responses and individuals not currently practicing as otorhinolaryngologists.

Data collection

The survey was conducted online via Survs.com, ensuring accessibility and convenience for participants. The Bulgarian National Society of Otorhinolaryngology, Head and Neck Surgery, distributed the survey via email to its members. The platform’s settings prevented multiple submissions from the same IP address. Questions covered demographic information, workplace distribution, perceived infection risks, psychological and financial impacts, and involvement in COVID-19-specific care. Responses were collected anonymously to encourage honest reporting using Survs.com methods.

Statistical analysis

Descriptive statistics summarize demographic data and workplace characteristics, including age distribution, years of experience, and practice settings. Crosstab analyses were employed to explore associations between key variables, such as perceived infection risk and workplace type. Statistical significance was assessed where applicable, and findings were presented using graphical representations to enhance clarity and interpretation. Data analysis, including descriptive statistics and cross-tabulations, was conducted using Survs.com with additional tests performed in Jamovi 2.3.28. The chi-square test of independence was conducted to assess the association between distinct categories. The chi-square statistic and corresponding p-value were calculated using the chi2_contingency function in Jamovi 2.3.28. Statistical significance was set at p-values <0.05. If the p-value was less than 0.05, the null hypothesis of no association between the variables was rejected.

## Results

**Figure 1:**
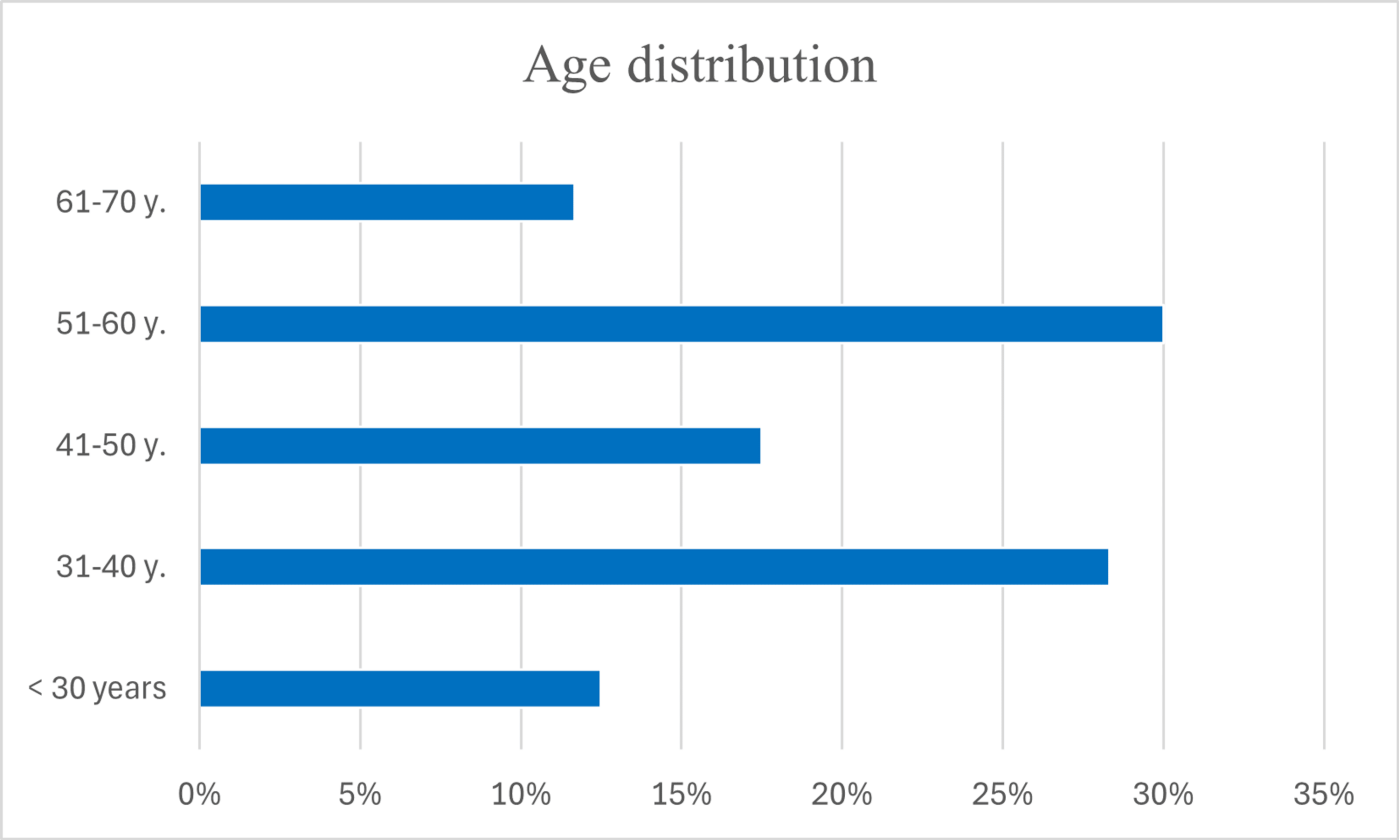
Age distribution of the respondents.

Of 241 invited participants, 120 completed the survey, yielding a response rate of 49.8%. The majority were experienced specialists, while a smaller proportion consisted of early-career professionals. The largest age group comprised 36 (30%) respondents aged 51-60 years, followed by 34 (28.3%) aged 31-40 years. The 41-50-year group included 21 (17.5%) respondents, while 15 (12.5%) were younger than 30 years. The smallest group, 14 (11.7%), consisted of specialists over 60 years old (Figure 1).

**Figure 2:**
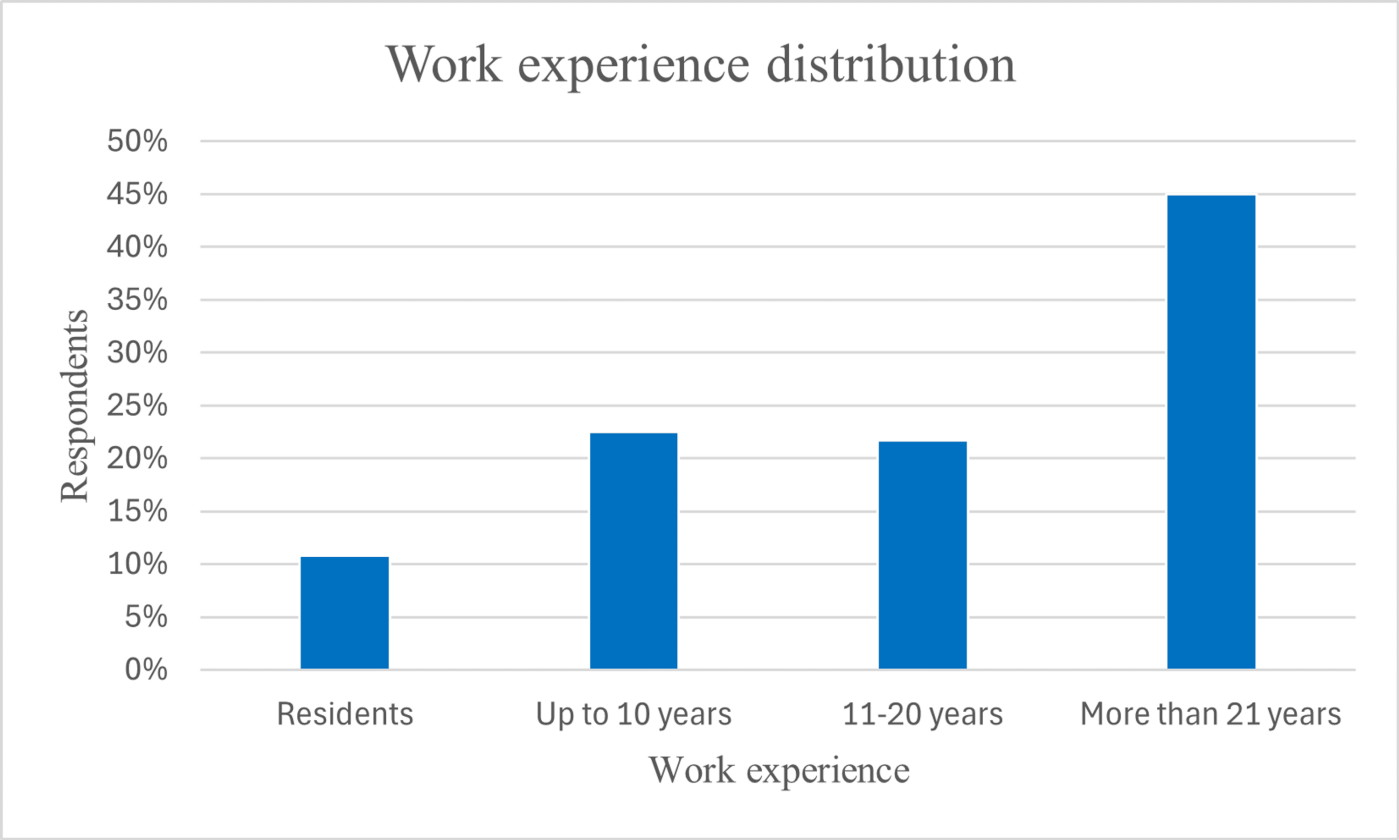
Work experience distribution of the respondents.

In terms of professional experience, 54 (45%) had more than 21 years of practice, while 27 (22.5%) had up to 10 years of experience, and 26 (21.7%) had 11 to 20 years of experience. Additionally, 13 (10.8%) were residents in training (Figure 2).

**Figure 3:**
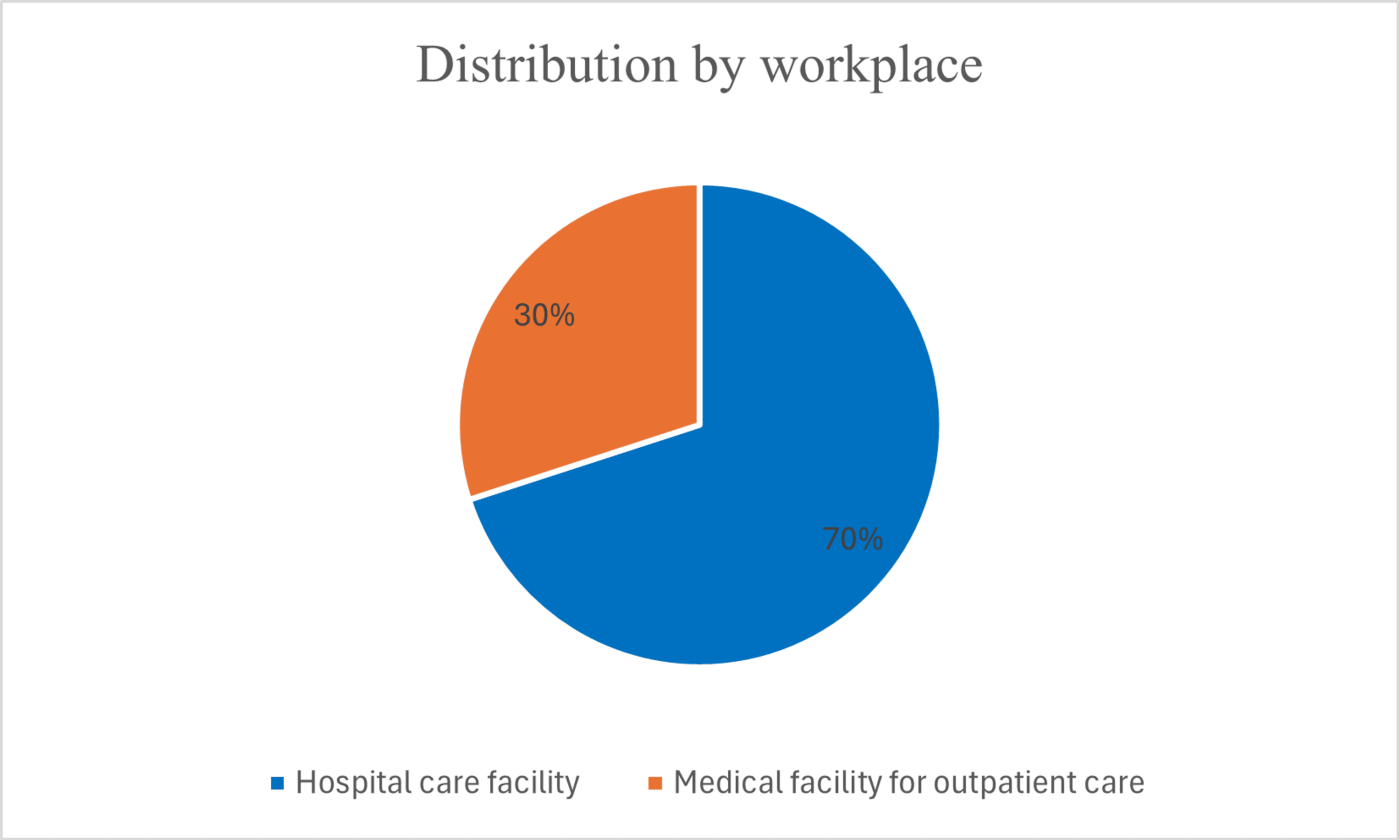
Workplace distribution of the respondents.

Among the surveyed otolaryngologists, 84 (70%) worked in hospital settings, while 36 (30%) practiced in outpatient medical institutions (Figure 3).

**Figure 4:**
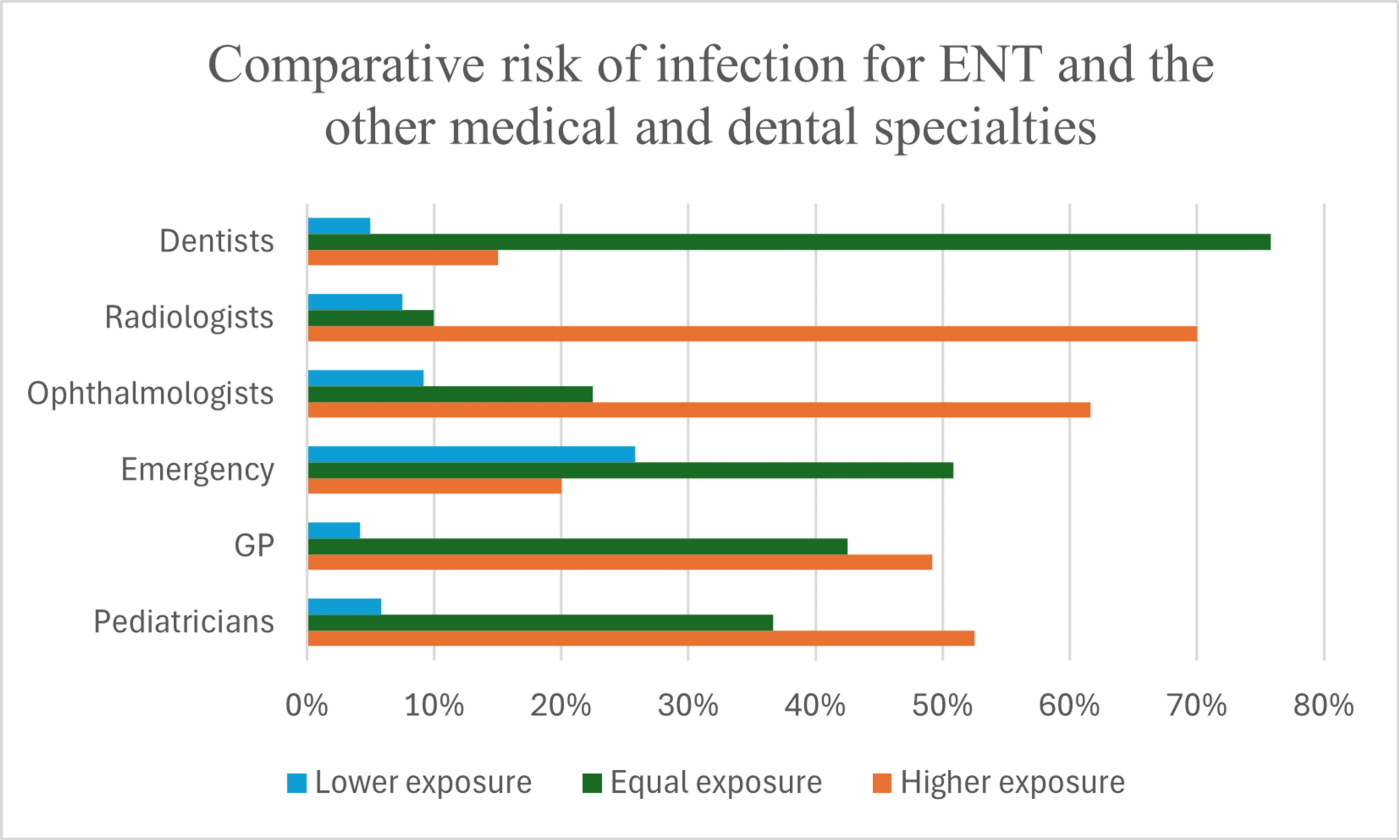
Comparative risk of infection with COVID-19 for ENT and other medical and dental specialities. ENT: ear, nose, and throat; GP: general practice

The perceived risk of COVID-19 infection was notably high, with 84 (70%) considering their risk higher than radiologists, 74 (61.6%) perceiving greater risk compared to ophthalmologists, and 63 (52.5%) and 59 (49.2%) believing their exposure exceeded that of pediatricians and general practitioners, respectively. While 61 (50.8%) felt their infection risk was comparable to emergency healthcare workers, 91 (75.8%) equated their exposure to that of dentists (Figure 4).

**Figure 5:**
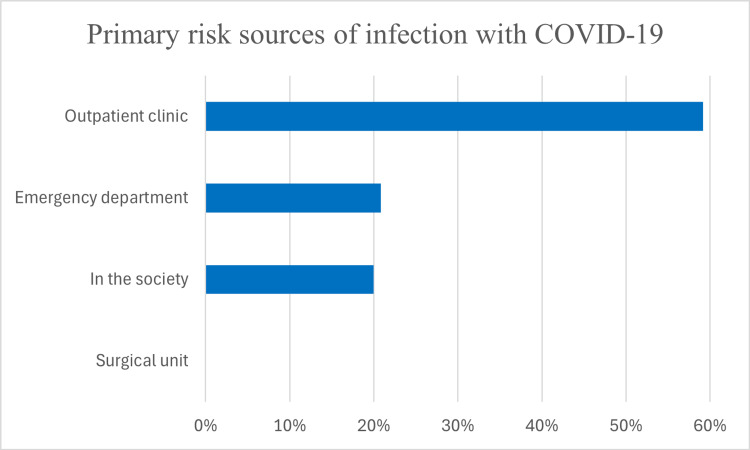
Primary risk sources of infection with COVID-19 according to respondents’ opinion.

Concerns regarding COVID-19 exposure were widespread, with 116 (96.6%) rating the probability of infection as “high risk,” while only 4 (3.3%) perceived the risk as low. Workplace exposure was identified as the primary source of infection by 95 (79.1%) of respondents, with 71 (59.1%) identifying outpatient clinics as the highest-risk settings, followed by 25 (20.8%) citing emergency departments and 24 (20%) pointing to community environments. Hospital surgical units were not regarded as a significant source of infection risk (Figure 5).

A chi-square test of independence showed a statistically significant association between the perceived risk of COVID-19 infection and work experience (χ² (3) = 14.25, p = 0.0026), suggesting that an otolaryngologist’s perception of infection risk varied significantly based on professional experience (Table 1).

**Table 1:** Characteristics of the statistical association between the perceived risk of COVID-19 infection and work experience. Significant differences (<0.05) in the categories between the perceived risk of COVID-19 infection and the work experience of otolaryngologists. n = number; χ² = chi-square test; df = degrees of freedom; p = probability

Perceived risk of COVID-19 infection	Work experience								Chi-square statistic, χ² (df)	P-value
	Group 1 (<10 years)		Group 2 (11–20 years)		Group 3 (>21 years)		Group 4 (residents)			
	n	%	n	%	n	%	n	%		
Low risk	4	3.3	0	0	0	0	0	0	14.25 (3)	0.0026
High risk	23	19.16	26	21.66	54	45	13	10.83		

**Figure 6:**
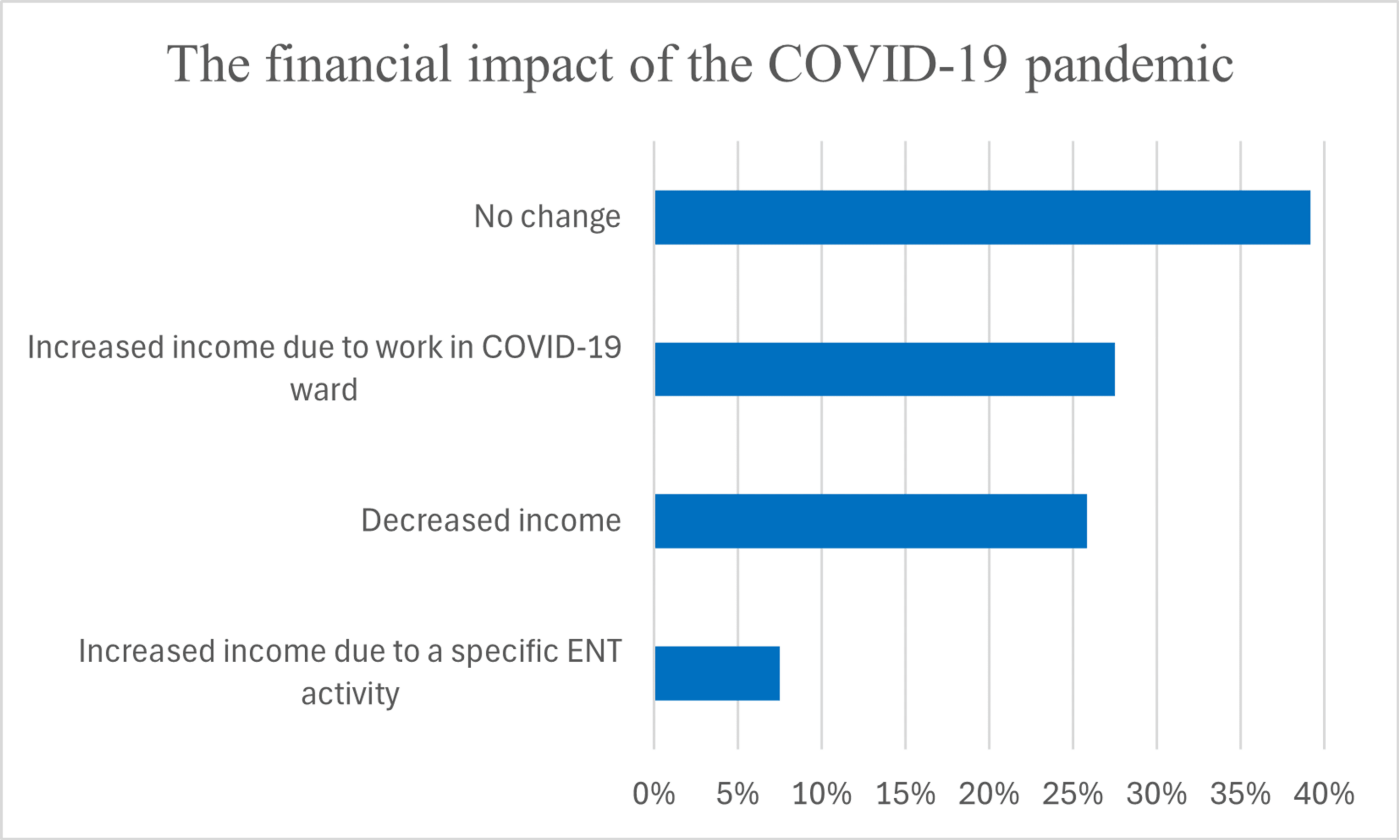
Distribution of the financial impact of the COVID-19 pandemic on otolaryngological practice in Bulgaria. ENT: ear, nose, and throat

The financial impact of the pandemic varied among respondents, with 47 (39.1%) reporting no change in income, 31 (25.8%) experiencing income reductions, and 42 (35%) reporting increased earnings. Among those with increased revenue, 33 (27.5%) attributed this to work in COVID-19 departments, while 9 (7.5%) reported growth related to ENT-specific activities (Figure 6).

A chi-square test indicated a statistically significant association between financial impact and work experience (χ² (9) = 44.0, p < 0.001) (Table 2).

**Table 2:** Characteristics of statistical association between the financial impact of the COVID-19 pandemic on otolaryngological practice and the work experience of otolaryngologists. Significant differences (<0.05) in categories between the financial impact of the COVID-19 pandemic on otolaryngological practice and the work experience of otolaryngologists. n = number; χ² = chi-square test; df = degrees of freedom; p = probability

Financial impact of the COVID-19 pandemic	Work experience								Chi-square statistic, χ² (df)	P-value
	Group 1 (<10 years)		Group 2 (11–20 years)		Group 3 (>21 years)		Group 4 (residents)			
	n	%	n	%	n	%	n	%		
Increased income due to ENT activity	0	0	0	0	9	7.5	0	0	44.0 (9)	<0.001
Increased income due to work in COVID-19 facilities	9	7.5	3	2.5	10	8.33	11	9.16		
Decreased income	3	2.5	9	7.5	18	15	1	0.83		
No change in income	15	12.5	14	11.66	17	14.16	1	0.83		
Total	27	22.5	26	21.67	54	45	13	10.83		

Income stability was reported most frequently by 47 (39.1%) respondents, primarily from work experience groups 1 (≤10 years) and 2 (11-20 years). A decrease in income was most prevalent among 31 (25.8%) respondents, particularly in group 3 (>21 years). Increased income from COVID-19 work was predominantly observed in groups 1 (≤10 years) and 4 (residents), while 9 (7.5%) in group 3 (>21 years) benefited from ENT-specific procedural shifts. These findings suggest that mid-career specialists faced greater financial strain, whereas younger professionals and those engaged in COVID-19-related work were more likely to experience income increases. A chi-square test of independence assessing the relationship between financial impact and workplace setting revealed a statistically significant association (χ² (3) = 11.68, p = 0.0086) (Table 3).

**Table 3:** Characteristics of statistical association between the financial impact of the COVID-19 pandemic on otolaryngological practice and the workplace setting. Significant differences (<0.05) in categories between the financial impact of the COVID-19 pandemic on otolaryngological practice and the workplace setting. n = number; χ² = chi-square test; df = degrees of freedom; p = probability

Financial impact of the COVID-19 pandemic	Workplace setting				Chi-square statistic, χ² (df)	P-value
	Hospital-based		Outpatient-based			
	n	%	n	%		
Increased income due to ENT activity	2	1.66	7	5.8	11.68 (3)	0.0086
Increased income due to work in COVID-19 facilities	24	20	9	7.5		
Decreased income	21	17.5	10	8.3		
No change in income	37	30.83	10	8.3		
Total	84	70	36	30		

Hospital-based otolaryngologists were more likely to report income reductions, with 65 (54.2%) experiencing financial decline compared to 22 (18.3%) in outpatient settings. In contrast, 20 (16.7%) hospital-based specialists reported no income change compared to 8 (6.7%) in outpatient settings.

Regarding direct involvement in COVID-19 care, 92 (76.6%) worked in COVID-19 wards, 6 (5.0%) in outpatient COVID-19 facilities, and 1 (0.8%) in a vaccination center. Additionally, 19 (15.8%) provided consultations or interventions in COVID-19 wards as needed, while 28 (23.3%) were not involved in COVID-specific care.

**Figure 7:**
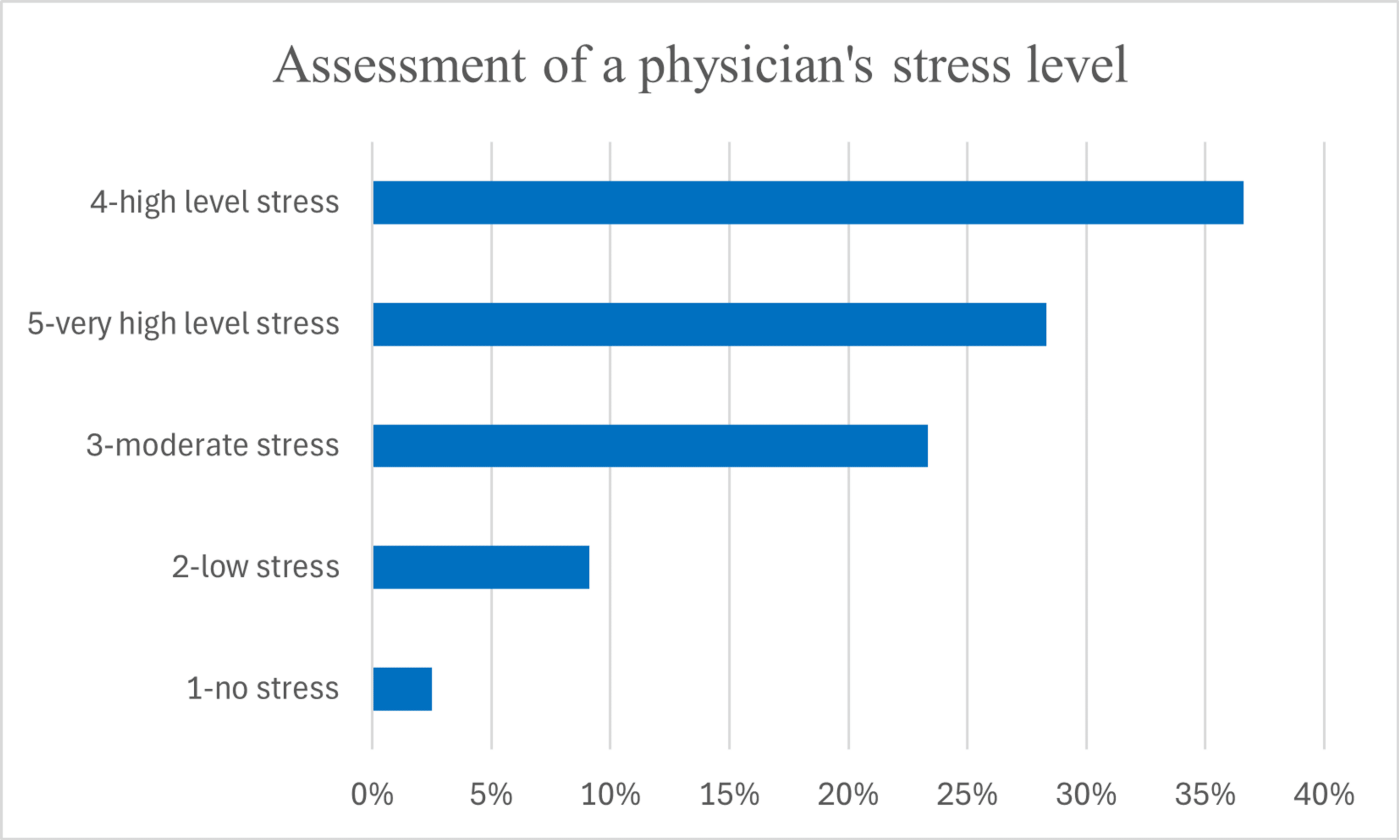
Assessment of a physician’s stress level.

The psychological impact of the COVID-19 pandemic was considerable, with 44 (36.6%) reporting high stress levels and 34 (28.3%) experiencing very high stress. A smaller group, 28 (23.3%), reported moderate stress, while 11 (9.1%) indicated little impact, and only 3 (2.5%) stated no effect on their mental health (Figure 7).

A chi-square test of independence examining stress level and work experience revealed a statistically significant association (χ² (12) = 22.9, p = 0.029) (Table 4).

**Table 4:** Characteristics of statistical association between stress level and work experience of the otolaryngologists. Significant differences (<0.05) in categories between stress level and work experience of the otolaryngologists. n = number; χ² = chi-square test; df = degrees of freedom; p = probability

Stress level	Work experience								Chi-square statistic, χ² (df)	P-value
	Group 1 (<10 years)		Group 2 (11–20 years)		Group 3 (>21 years)		Group 4 (residents)			
	n	%	n	%	n	%	n	%		
1: No stress	0	0	0	0	2	1.67	1	0.83	22.9 (12)	0.029
2: Low stress	0	0	2	1.67	9	7.5	0	0		
3: Moderate stress	12	10	3	2.5	12	10	1	0.83		
4: High stress	6	5	13	10.83	18	15	7	5.83		
5: Very high stress	9	7.5	8	6.67	13	10.83	4	3.33		
Total	27	22.5	26	21.67	54	45	13	10.83		

The analysis also identified a statistically significant relationship between stress levels and workplace setting (χ² (4) = 12.4, p = 0.015), indicating that the work environment significantly influenced the stress experienced during the pandemic (Table 5).

**Table 5:** Characteristics of statistical association between stress level and workplace settings. Significant differences (<0.05) in categories between stress level and workplace settings. n = number; χ² = chi-square test; df = degrees of freedom; p = probability

Stress level	Workplace settings				Chi-square statistic, χ² (df)	P-value
	Hospital-based		Outpatient-based			
	n	%	n	%		
1: No stress	3	2.5	0	0	12.4 (4)	0.015
2: Low stress	3	2.5	8	6.66		
3: Moderate stress	21	17.5	7	5.83		
4: High stress	34	28.33	10	8.33		
5: Very high stress	23	19.16	11	9.16		
Total	84	70	36	30		

Among hospital-based otorhinolaryngologists, 34 (28.3%) were in category 4 (high stress), while 23 (19.2%) were in category 5 (very high stress). Among outpatient-based specialists, the largest group, 11 (9.2%), were in category 5 (very high stress), while 10 (8.3%) were in category 4 (high stress).

## Discussion

These findings highlight the profound professional, financial, and psychological challenges otolaryngologists faced during the COVID-19 pandemic, providing valuable insight into their experiences across diverse clinical settings. These results align with observed global trends but reveal unique regional aspects that warrant deeper analysis.

Otolaryngologists are among the specialties at the highest risk of contracting COVID-19 due to direct contact with the upper respiratory tract, where the viral load can be substantial [9,10]. The survey reveals that over 60% of Bulgarian ENT specialists consider their risk to be higher than radiologists and ophthalmologists. Notably, early warnings about COVID-19 came from Dr. Li Wenliang, an ophthalmologist, who later succumbed to the disease. This underscores the risk faced by specialists working in close proximity to the upper respiratory tract [11-13]. A majority of respondents, 91 (75.8%), equated their risk to that of dentists, a group widely recognized for its heightened exposure risk due to aerosol-generating procedures [14-16]. Studies have shown that ENT procedures involving the nasal cavity, pharynx, and ear carry risks due to droplet generation [17-19]. The fact that routine ENT examinations are perceived as high risk emphasizes the need for protocols to reduce exposure during essential examinations. Our analysis demonstrated no statistically significant association between workplace type and perceived risk of infection among otolaryngologists during the COVID-19 pandemic. This uniformity in risk perception may reflect the inherently high-risk nature of the otolaryngological practice, irrespective of the workplace setting, due to close patient interactions and aerosol-generating procedures. The results suggest that standardized infection control protocols and safety measures likely contributed to the constant perception of risk. Future studies could explore additional factors influencing risk perception, such as variations in protective equipment availability, individual experiences, or differences in institutional policies. In response, otolaryngologists globally have advocated for new infection control guidelines, including the permanent use of higher-grade personal protective equipment (PPE) (e.g., KN95 or FFP2 masks) and even modified examination techniques to mitigate exposure risk [18]. Adopting these protocols in Bulgarian practices could align local standards with those emerging internationally. The statistically significant relationship between perceived infection risk and work experience suggests that professional background influences risk perception. More experienced otolaryngologists may have recognized a greater occupational hazard due to their prolonged exposure to patients and a higher likelihood of performing aerosol-generating procedures. This finding aligns with previous studies indicating that specialists with extensive clinical experience tend to have a heightened awareness of occupational risks.

The survey’s findings indicate substantial psychological strain. A significant proportion of respondents, 78 (65%), experienced high or very high stress levels. This is consistent with studies on healthcare workers’ mental health during the pandemic, which highlight high rates of burnout, anxiety, and depression among frontline medical staff [20]. ENT specialists, particularly in countries with limited resources and PPE, might be at an elevated risk of psychological distress due to both the direct threat of infection and concerns over inadequate protective measures. Most Bulgarian otolaryngologists, 106 (88.2%), felt under moderate to very high stress levels. None of them reported undergoing formal psychological stress assessments. Only two respondents had undergone such research but had not followed any stress-reducing measures from the institution they worked for. These findings emphasize the need for broad-based approaches to stress mitigation, addressing the collective challenges faced by healthcare professionals due to COVID-19. The relationship between stress levels and work experience was statistically significant, suggesting that mid-career and senior professionals experienced greater psychological distress. This could be due to the increased professional responsibility placed on experienced specialists, who often had to balance clinical duties, administrative roles, and the challenges of adapting to rapidly changing protocols. Workplace settings also significantly influenced stress levels, with hospital-based specialists reporting higher stress compared to those in outpatient settings. The increased exposure to COVID-19 patients, greater workload, and heightened procedural risks may have contributed to this disparity. These findings align with previous research demonstrating that frontline healthcare workers, particularly those in hospital environments, experienced higher stress and burnout levels during the pandemic. The need for targeted mental health interventions, particularly for hospital-based ENT specialists, is evident from these results. The psychological strain of working in high-risk environments can have enduring consequences, such as reduced job satisfaction and diminished quality of care. To mitigate these effects, it is essential to implement targeted mental health interventions and adjust workloads to support healthcare workers effectively.

During pandemics or epidemics, healthcare workers often face considerable stress due to the nature of their work. For instance, during the 2014-2015 Ebola outbreak in Sierra Leone, healthcare workers in direct contact with patients experienced various psychological challenges, including obsessive-compulsive tendencies, paranoia, heightened interpersonal sensitivity, and depression [21]. Similarly, the 2003 SARS epidemic significantly affected the mental health of healthcare workers, with studies reporting emotional distress in up to 57% of healthcare workers [22]. In particular, no specific data regarding ENT specialists was observed in either study.

Although otolaryngologists were regarded as a high-risk group for contracting COVID-19 due to the nature of their procedures, a study by Walvik et al. (2021) in Denmark found no participants showing severe symptoms of depression or anxiety during the survey period [23]. This finding can be attributed to several factors, i.e., Denmark experienced a relatively controlled pandemic, the supply of PPE had stabilized, and COVID-19 testing was widely accessible.

The financial impact of the pandemic was another area of notable variation. While 47 (39.1%) respondents reported no change in income, 31 (25.8%) experienced financial reductions, particularly among those with more than 21 years of experience. This decline may be attributed to the temporary suspension of elective procedures, a key source of revenue for ENT specialists. In contrast, younger professionals and residents were more likely to report increased income, largely due to their engagement in COVID-19-specific work, including shifts in COVID-19 wards and outpatient facilities. The statistically significant association between financial impact and work experience highlights the uneven economic burden of the pandemic across different career stages.

The study’s findings underscore the wide-ranging impact of the pandemic on otolaryngological practice. The significant associations between perceived infection risk, financial outcomes, psychological stress, and work experience or workplace setting highlight the multifaceted challenges faced by ENT specialists. These insights emphasize the necessity for tailored support measures, including financial relief programs, improved infection control policies, and mental health interventions, to mitigate the long-term effects of the pandemic on the specialty.

While this study provides valuable data, certain limitations must be acknowledged. Factors such as local infection rates, hospital workload, and access to PPE may also contribute to stress levels and risk perception, which were not controlled for in this study. The survey was conducted within a single country, and findings may not be fully generalizable to other healthcare systems. Additionally, self-reported data may be subject to response bias, particularly in the assessment of stress levels, financial impact, or perceptions of infection risk. Nevertheless, the diversity within the respondent group, including varying levels of work experience, age, and practice settings, adds depth to the findings. To enhance the robustness and generalizability of these results, future studies should aim to include a larger, more representative sample and consider longitudinal designs to track the evolving impacts of the pandemic over time. Despite these limitations, the study offers significant insights that can inform policies, protective measures, and support systems for otolaryngologists during and beyond public health crises.

## Conclusions

A combination of heightened infection risk, significant practice adaptations, and psychological strain demonstrates the impact of the COVID-19 pandemic on otolaryngology practice in Bulgaria. The results of this survey illustrate the sweeping changes experienced by Bulgarian otolaryngologists during the COVID-19 pandemic, from heightened infection risk to increased psychological burden. These findings align with international trends yet reveal unique local characteristics that require tailored responses. Ensuring PPE availability, addressing diagnostic delays, and supporting mental health are critical to fortifying the resilience of otolaryngology in future health crises. This study contributes valuable insights to the ongoing conversation around pandemic preparedness and the future of ENT practice in Bulgaria and beyond, as well as potential policy adjustments to address these challenges in future public health crises.
